# Tracking HIV-1-Infected Cell Clones Using Integration Site-Specific qPCR

**DOI:** 10.3390/v13071235

**Published:** 2021-06-25

**Authors:** Leah D. Brandt, Shuang Guo, Kevin W. Joseph, Jana L. Jacobs, Asma Naqvi, John M. Coffin, Mary F. Kearney, Elias K. Halvas, Xiaolin Wu, Stephen H. Hughes, John W. Mellors

**Affiliations:** 1 Department of Medicine, University of Pittsburgh, 3550 Terrace Street, Scaife Hall-818, Pittsburgh, PA 15261, USA; ldb43@pitt.edu (L.D.B.); kej38@pitt.edu (K.W.J.); jlj90@pitt.edu (J.L.J.); anaqvi@pitt.edu (A.N.); ekh2@pitt.edu (E.K.H.); 2 Cancer Research Technology Program, Leidos Biomedical Research, Inc., 8560 Progress Drive, ATRF, Room C3004, Frederick, MD 21701, USA; shuang.guo@nih.gov (S.G.); forestwu@mail.nih.gov (X.W.); 3 Department of Molecular Biology and Microbiology, Tufts University, 145 Harrison Avenue, Jaharis 409, Boston, MA 02111, USA; john.coffin@tufts.edu; 4 HIV-Dynamics and Replication Program, National Cancer Institute, 1050 Boyles Street, Building 535, Room 308, Frederick, MD 21702, USA; kearneym@mail.nih.gov (M.F.K.); hughesst@mail.nih.gov (S.H.H.)

**Keywords:** HIV-1 reservoir, HIV-1-infected cell clones, proviral integration sites, repliclones

## Abstract

Efforts to cure HIV-1 infection require better quantification of the HIV-1 reservoir, particularly the clones of cells harboring replication-competent (intact) proviruses, termed *repliclones*. The digital droplet PCR assays commonly used to quantify intact proviruses do not differentiate among specific repliclones, thus the dynamics of repliclones are not well defined. The major challenge in tracking repliclones is the relative rarity of the cells carrying specific intact proviruses. To date, detection and accurate quantification of repliclones requires in-depth integration site sequencing. Here, we describe a simplified workflow using integration site-specific qPCR (IS-qPCR) to determine the frequencies of the proviruses integrated in individual repliclones. We designed IS-qPCR to determine the frequencies of repliclones and clones of cells that carry defective proviruses in samples from three donors. Comparing the results of IS-qPCR with deep integration site sequencing data showed that the two methods yielded concordant estimates of clone frequencies (*r* = 0.838). IS-qPCR is a potentially valuable tool that can be applied to multiple samples and cell types over time to measure the dynamics of individual repliclones and the efficacy of treatments designed to eliminate them.

## 1. Introduction

HIV-1 infection is controlled, but not cured, by antiretroviral therapy (ART) because long-lived CD4+ T cells carry replication-competent (intact) proviruses. These infected cells can clonally expand in response to antigen-driven or homeostatic stimulation, or rarely, as a consequence of the provirus integrating in one of seven oncogenes [1,2,3]. Clones of infected cells can arise early after an individual is infected and persist for many years after initiation of ART [4]. Most (95–98%) proviruses in individuals on long-term ART are defective, having large deletions or other lethal mutations [5,6,7,8,9]. A small subset of infected clones carry intact proviruses that can produce low-level viremia on ART [10,11] and may contribute to rebound viremia if ART is stopped [12,13,14,15]. Clones of cells that carry intact infectious proviruses, termed *repliclones*, are an important component of the HIV-1 reservoir and may constitute most of it. Accordingly, it is important to accurately measure the effects of long-term ART and experimental interventions intended to reduce the sizes of the HIV-1 reservoir to determine what fraction of repliclones respond to the intervention [11].

The challenge in determining the sizes of clones of infected cells lies in accurately determining, in samples from HIV-1-positive donors, the number of proviruses integrated at exactly the same position in the human genome [16]. In addition, matching integration sites to full-length intact proviruses is a critical step in differentiating repliclones from clones carrying defective proviruses: most PCR methods used to analyze HIV-1 proviruses provide only a partial sequence of the provirus. Having two proviruses that are identical in one region does not necessarily mean the proviruses are identical; there may be differences in portions of the proviruses that were not analyzed [17,18]. The first identification of the integration site of an intact provirus in an expanded clone (called AMBI-1) was a breakthrough in identifying a full-length provirus that produced infectious virus found in plasma [10]. More recently, several groups have described methods that can be used to characterize full-length proviruses and their corresponding integration sites [18,19,20,21]. Although these approaches represent an important advance in characterizing the HIV-1 reservoir, they are limited by the small number of proviruses that can be characterized relative to the size and complexity of the reservoir.

Other approaches that have been used to measure the HIV-1 reservoir involve quantitative viral outgrowth (QVOA) [22,23,24] and the more sensitive droplet digital quantification of intact proviral DNA (IPDA) [9,25]. Although informative, these methods are not able to readily quantify the sizes or the in vivo dynamic behavior of individual clones of HIV-1-infected cells [26]. Here, we present an integration site-specific qPCR assay (IS-qPCR) that can be used to measure the sizes of specific clones of infected cells. The IS-qPCR assay requires that the exact location and orientation of the provirus be known [11,19,20,21]. With this information, IS-qPCR primers and probes are designed and used to selectively amplify, and detect by probe hybridization, the host–virus junctions of the proviruses in clones of interest. Here, we describe the IS-qPCR method and compare its results to the results obtained from a deep integration site assay (ISA) dataset (10^3^–10^4^ sites per sample) [11,27,28].

## 2. Materials and Methods

### 2.1. Isolation and Extraction of Cells

ACH-2 cells were obtained from the NIH AIDS Reagent Program. Peripheral blood mononuclear cells (PBMC) were obtained from volunteers who gave written informed consent for study participation. All participants were enrolled at the University of Pittsburgh Clinical Trials Unit between April of 2014 and June of 2015. Total CD4+ T cells were purified from PBMC using negative selection with the EasySep™ Human CD4+ T cell Isolation Kit (STEMCELL Technologies, Vancouver, Canada). Cells were initially lysed and the cell lysate was sonicated according to Hong et al. [29]. Genomic DNA was extracted from ACH-2 cells and PBMC and/or total CD4+ T cells using the King Fisher Flex (Thermo Fisher Scientific, Waltham, MA, USA) with the MagMAX DNA Multi-Sample Ultra 2.0 Kit according to the manufacturer’s instructions. Nucleic acid was quantified with Quanti-iT™ PicoGreen^®^ dsDNA reagent on a SpectraMax i3x (Molecular Devices, LLC, San Jose, CA, USA).

### 2.2. Identification of Integration Sites from Clones of Interest

Integration sites for proviruses of interest were identified as previously described [11,19,20]. In brief, the proviral endpoint of extracted genomic DNA derived from PBMC and/or total CD4+ T cells was determined by Poisson distribution statistics using single-genome sequencing of HIV-1 amplicons containing a portion of *gag (p6)*, all of *pro*, and the first 900 bases of *pol* (*gag-pro-pol)* [11,30]. Genomic DNA extracted from the samples was diluted across a microtiter plate such that each well contained, on average, less than 1 provirus. Whole-genome amplification of the cellular DNA was then performed using an in-house workflow using multiple displacement amplification (MDA) [31,32] and a specificity-enhancing linker-mediated PCR that amplifies across the 5′ LTR host–virus junction [11,20,21], or by the method described by Patro et al. [19] (Supplementary Table S7). An aliquot of the MDA product was subsequently screened by *gag-pro-pol* or near full-length proviral amplification [33] and sequencing to identify the well(s) that contained the proviruses of interest [20,21]. The integration sites for the proviruses were determined using the MDA-amplified DNA from wells of interest as reported [11,20,21,28]. We then confirmed the structure and integration site of each provirus by amplifying the entire provirus and flanking host region using (host-to-full-length provirus-to-host (HFH)) PCR from extracted genomic DNA without further modification. Two overlapping PCR amplicons were generated for each provirus by HFH and sequenced [11].

### 2.3. Primer and Probe Design

For each provirus of interest, we designed a forward PCR primer immediately upstream (26–113 bp) of the 5′ host–virus junction. The reverse PCR primer was designed to match sequences immediately downstream (24–186 bp) of the host–virus junction in U3. For the qPCR probe, we selected a region spanning the host–virus junction, determined as described above, to ensure a highly-specific target. The probe was labeled with a 5′ 6-FAM fluorophore, a 3′ dark quencher, and an additional internal ZEN quencher (IDT, USA). Primer and probe sequences are provided in Supplementary Table S1. The size of the amplicons generated for each provirus ranged from 110 to 225 bp in length. We confirmed that the correct target sequence was amplified by dideoxy chain termination sequencing (Sanger) of the PCR products and cross-referencing the amplicon sequences with those determined by HFH and sequencing [11,20,21].

### 2.4. Preparation of Standards for DNA Quantification

The qPCR standard for each of the proviruses was prepared by PCR amplification of the host–virus junction (5′ LTR) using host primers [11] paired with an HIV-1-specific reverse primer that matched sequences in R [33]. The qPCR standards for the clones with defective proviruses were prepared by PCR amplification using reported primers [20,21]. The amplifications were performed using RANGER Mix (Meridian Bioscience, Inc., Cincinnati, OH, USA) in 10 µL reactions, where 2 µL of the DNA extract was added to 8 µL master mix according to the manufacturer’s suggested proportions. PCR conditions were as follows: 95 °C for 3 min, 98 °C for 10 s, and 60–61 °C for 45 s for 30 cycles. The PCR product was visualized on a 0.7–1% agarose gel in sodium borate buffer and pooled for purification, first using the GeneJET Gel Extraction Kit (Thermo Fisher Scientific, Waltham, MA, USA), followed by KAPA Pure Beads (Kapa Biosystems, Wilmington, MA, USA), according to the manufacturers’ instructions. The copy number was estimated on the basis of DNA quantification via Quanti-iT™ PicoGreen^®^ dsDNA reagent on a SpectraMax® i3x (Molecular Devices, LLC, San Jose, CA, USA) and the Thermo Fisher DNA Copy Calculator. Serial endpoint dilution of the standard was used to validate the expected copy number as determined by Poisson statistics (62% of replicates positive at dilution to 1 copy/reaction). Once quantified, the DNA standard was diluted to 1000 copies/µL in 5 mM Tris-HCl (pH 8.0) (Invitrogen Corporation, Carlsbad, CA, USA) and stored at −20 °C in single-use aliquots.

### 2.5. Quantification of Individual Proviruses by IS-qPCR

Total nucleic acids were extracted from cells and diluted to a concentration of ≤70 ng/µL to prevent inhibition of PCR by excess nucleic acid. For qPCR, 10 µL of the nucleic acid extract was used in a total volume of 25 µL containing 400 nM forward and reverse primers and 200 nM probe in ready-made LightCycler^®^ 480 Probes Master mix (Roche Molecular Systems, Inc., Pleasanton, CA, USA). Cycling parameters for qPCR were 95 °C for 5 min, followed by 95 °C for 15 s and 60 °C for 1 min, for 50 cycles of amplification. Sample concentrations were derived from within-run standard curves created by serial 3.16-fold dilution of the integration site-specific standard, described above, ranging from 3000 to 3 copies per well. Values reported below the 3 copy threshold were extrapolated from the linear within-run standard curve. Quantification of the copies of the specific provirus in the sample was determined using LightCycler^®^ 480 software version 1.5.1.62 (Roche Diagnostics Corporation, Indianapolis, IN, USA). Graphic representations of the frequencies of the specific proviruses and statistical analyses were performed in GraphPad Prism version 8.0.0 for Windows (GraphPad Software, San Diego, CA, USA).

To determine the number of cell equivalents assayed in a PCR reaction, the amount of genomic DNA in the nucleic acid extract was quantified in duplicate or triplicate using qPCR for the CCR5 gene, as reported [29,34]. CCR5 copy numbers were used to calculate the number of copies of a specific provirus per 1 million cell equivalents. The total number of proviruses in the nucleic extract was quantified in duplicate or triplicate using qPCR for the R/U5 region of LTR. LTR primers with modified degenerate bases [35] (LGC Biosearch Technologies, Middleton, WI, USA) (Supplementary Table S2) were designed to amplify LTRs that have single-nucleotide polymorphisms at known locations (analysis from Los Alamos National Laboratory HIV Sequence Database). These primers were used in tandem with a minor groove binder (MGB) modified probe (Thermo Fisher Scientific, Waltham, MA, USA) (Supplementary Table S2) using the same reaction and cycling parameters for qPCR provided above. Copies of an LTR standard were quantified using a within-run standard corresponding to position 87 through 8680 (HXB2 numbering) constructed by amplification of proviral DNA from the JLat 6.3 cell line (NIH AIDS Reagent Program). To calculate the number of proviruses in a sample, the measured number of LTR copies was divided by 2 to account for two LTR copies per provirus. Clone frequencies were calculated using the average number of proviruses of interest and the average number of total proviruses. IS-qPCR was assayed in multiple assays per sample with each assay consisting of triplicate PCR reactions. For PBMC samples, both the specific proviruses and total proviruses were each adjusted per 1 million CD4+ T cells using the percentage of PBMC that were CD3/CD4+ T cells as previously reported for each donor [11]. Intra-assay variability from IS-qPCR assays are reported in Supplementary Table S4 along with the mean and standard deviation across assays.

### 2.6. Deep Integration Site Analysis

A detailed methodology of the linker-mediated PCR and bioinformatics pipeline used for deep integration site analysis has been described in [27,28]. In brief, genomic DNA is fragmented, the ends are repaired, and a single dA is added to the 3′ ends of the fragmented DNA to which a T-linker adapter is ligated. A nested PCR amplification is performed using a LTR-specific primer and a primer in the adaptor to selectively amplify the host–virus junction. Illumina adaptors and indices are added during the nested PCR step. The Illumina sequencing data are analyzed through a pipeline to demultiplex, trim, and filter those sequences with an LTR–genome junction. After excluding the viral sequences in the reads, the sequences are mapped to the human genome Hg19 using the BLAT alignment tool.

### 2.7. Sequence Alignments and Mapping

Full-length HIV-1 proviral sequences were aligned using a pairwise MUSCLE alignment in CLC Genomics Workbench version 20.0.4 (QIAGEN Digital Insights, Aarhus, Denmark). Annotations and visualization were based on the genomic region coordinates and HIV-1 Gene Map of HXB2, provided by the Los Alamos HIV Database (https://www.hiv.lanl.gov/content/sequence/HIV/MAP/landmark.html, accessed on 30 December 2020).

## 3. Results

### 3.1. IS-qPCR Workflow

**Figure 1:**
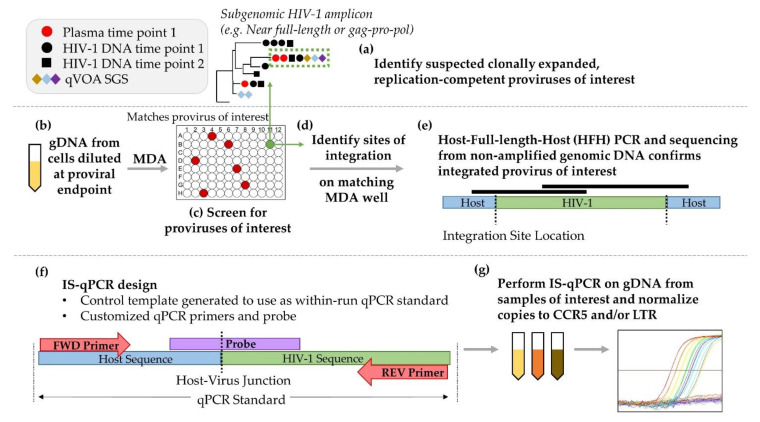
IS-qPCR workflow. (**a**) Proviruses whose sequences matched *gag-pro-pol* RNA sequences from plasma and quantitative viral outgrowth assays (QVOA) were analyzed as described previously [11,30]. (**b**) Genomic DNA (gDNA) was diluted to a proviral endpoint, amplified by MDA and (**c**) screened (e.g., near-full-length or *gag-pro-pol* PCR) for the proviruses of interest. (**d**) The corresponding MDA product was analyzed to determine the integration site and orientation of the provirus. (**e**) The sequence and integration sites of proviruses of interest were confirmed by PCR amplification and sequencing of the full-length proviruses as described [11]. (**f**) Host and HIV-1 primers flanking the integration site were designed to amplify a ~150 bp amplicon and a probe spanning the host–virus junction was prepared. A within-run quantification standard for each provirus was generated by amplifying a sequence (<700 bp) spanning the host–virus junction from genomic DNA. (**g**) IS-qPCR analysis using gDNA from sample(s) of interest.

We developed an integration site-specific qPCR assay (IS-qPCR) that targets the host–virus junctions of proviruses in clonally expanded cells. Figure 1 shows the workflow, modified from [11,19], for identifying the host–virus junctions of the proviruses in repliclones that we subsequently quantified by IS-qPCR. Reagents and resources used in the IS-qPCR workflow are listed in Supplementary Table S7. We first analyzed single-genome sequencing data from viral RNA in plasma for the region encoding HIV-1 *gag-pro-pol*, from HIV-1 DNA in PBMC and from viral RNA in supernatants in p24-positive quantitative viral outgrowth assay (QVOA) wells to identify suspected repliclones (Figure 1a) [11,30]. Next, genomic DNA was diluted across a microtiter plate such that each well contained, on average, less than 1 *gag-pro-pol* containing provirus (Figure 1b). Multiple displacement amplification (MDA) was performed on each of the wells to non-specifically amplify the genomic DNA [11,19,20,21]. An aliquot of the MDA product was subsequently screened by *gag-pro-pol* or near full-length proviral amplification and sequencing [33] to identify the well(s) that contained the proviruses of interest (Figure 1c) [11,19,20,21]. The integration sites for the proviruses were determined (Figure 1d) using the MDA-amplified DNA from wells of interest as reported [11,20,21,28]. After identifying the integration site and the orientations of the proviruses, forward PCR primers that matched the host sequences adjacent to the 5′ LTR of the provirus were designed using the human genome assembly reference Hg19 sequence from the UCSC Human Genome Browser [36,37]. We confirmed the identity of each of the proviruses in non-amplified genomic DNA (Figure 1e) by generating two overlapping PCR amplicons using primers that matched host sequences paired with primers that matched HIV-1 sequences, which also confirmed the specificity of the host–virus junctions (Figure 1f).

For each IS-qPCR assay, we designed primers flanking the integration site and a probe spanning the host–virus junction. A smaller (<700 bp) product containing the host–virus junction was amplified, purified, quantified, and used as a within-run qPCR standard specific for each provirus. Replicates of genomic DNA were assayed in triplicate by IS-qPCR and the results were normalized for total proviruses in the sample by replicate LTR qPCR, assuming two LTRs per provirus, and for cell equivalents by replicate qPCR for CCR5, assuming two copies per cell (Figure 1g). Clones with defective proviruses were characterized and quantified in the same way, except plasma or QVOA well sequences were not available for matching.

### 3.2. Validation of IS-qPCR Using the Major ACH-2 Provirus Integrated in the NT5C3A Gene

As a proof of concept, IS-qPCR was tested using genomic DNA extracted from ACH-2 cells. The HIV-1-infected ACH-2 T cell line has been reported to have one predominant replication-competent provirus [38]. The integration site of the predominant provirus has been reported to be in the NT5C3A gene [39,40,41], which allowed us to bypass the first four steps in the workflow. ACH-2 cells have also been shown to harbor other integrated proviruses in lower abundance, which is consistent with there being ongoing replication of HIV-1 in the cell line [41]. We used ACH-2 cells to evaluate the performance of IS-qPCR for the provirus integrated in the NT5C3A gene.

Genomic DNA from ACH-2 cells was extracted and the concentration determined using Quanti-iT™ PicoGreen^®^ dsDNA reagent on a SpectraMax i3x (Molecular Devices, LLC, San Jose, CA, USA). Cell equivalents were estimated by qPCR for CCR5. Serial dilutions of ACH-2 DNA containing 100, 30, 10, 3, 1, and 0.3 cell equivalents were assayed by IS-qPCR. Table 1 shows the average number of cell equivalents and copies of the NT5C3A provirus (over three independent runs) detected in the assay. The results show that the number of copies of the NT5C3A provirus detected were similar to the cell equivalents assayed across a broad range of cell inputs. 

### 3.3. Application of IS-qPCR of Clones in Clinical Samples

**Figure 2:**
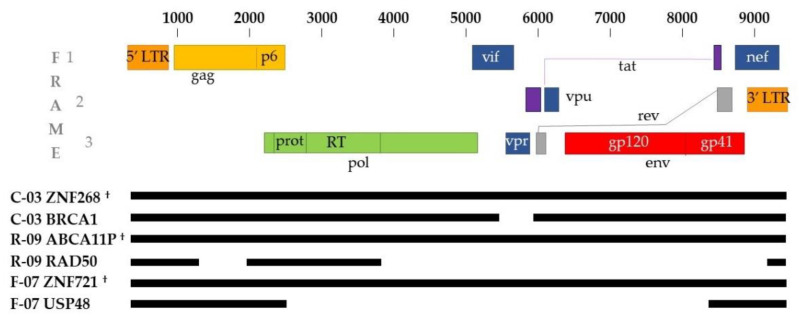
Alignments of the sequences of the HIV-1 proviruses in clonally expanded cells that were used to validate the IS-qPCR assay. Sequences were aligned using MUSCLE pairwise alignment. Black lines represent the sequences obtained for each of the proviruses and blank spaces represent deletions. Sequences are labeled with a letter and number designation to indicate the donor who was the source of the clone followed by the name of the gene in which the provirus was integrated. Intact infectious proviruses are denoted by ^†^.

In a previous study, large clones of HIV-1-infected cells that carried intact proviruses (called *repliclones*) were found, using methods illustrated in Figure 1a, to be the source of non-suppressible viremia on ART [11]. Donor demographics, clinical histories, immunologic and virologic characteristics are listed in Supplementary Tables S5 and S6. The integration sites of the proviruses in the repliclones were determined and the full-length proviruses were amplified and sequenced. The frequencies of each of the repliclones were also determined using deep integration site analysis (ISA) [11,27,28]. To validate IS-qPCR in clinical samples, we designed specific IS-qPCR assays for three of the well-characterized repliclones and analyzed the frequencies of repliclones from the same samples that were used to obtain the deep ISA data (Table 2 and Supplementary Table S3). We also obtained host–virus junctions and near full-length sequences from clones with defective proviruses from each of these individuals as described [20,21]. The structures of each of the proviruses in the six clones quantified by IS-qPCR are shown in Figure 2.

**Figure 3:**
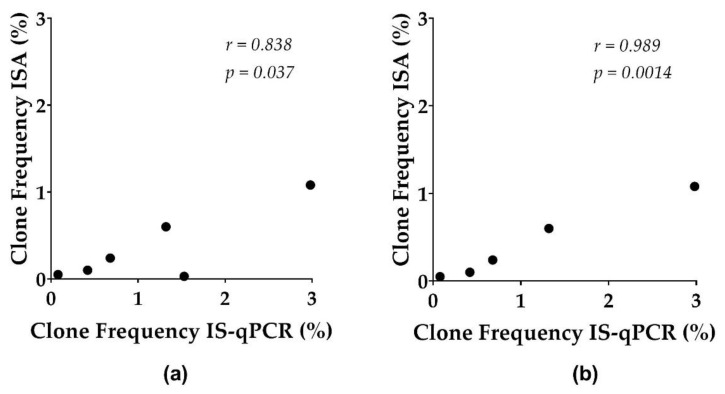
Strong positive correlation between clone frequencies (%) estimated by IS-qPCR and ISA for (**a**) all proviruses of interest, and (**b**) excluding provirus R-09 ABCA11P (apparent outlier). The Pearson correlation (*r*) and two-tailed *p* value were computed using the clones frequencies reported in Table 2.

To ensure adequate sampling, we assayed over 1 million cell equivalents (1.3–6.2 million cells) in 2–16 replicates (each replicate consisting of 3 PCR reactions) for PBMC and/or total CD4+ T cells (see Supplementary Tables S3 and S4). We evaluated the sensitivity and accuracy of IS-qPCR for the proviruses in each of the clones and compared the IS-qPCR data with deep ISA data obtained from the same samples (Table 2). Data from both IS-qPCR and qPCR quantifying total proviruses were adjusted to 1 million CD4+ T cells [11]. We report the frequencies of the clone from IS-qPCR as the percentage of the average number of proviruses detected for a specific clone divided by the average number of total proviruses detected in multiple samples obtained from the same donor (Supplementary Table S3). The frequencies reported in Table 2 are the averages across all samples tested for each donor. Intra-assay variability is reported in Supplementary Table S4. The calculated frequencies of proviruses determined by IS-qPCR were comparable to the frequencies from the deep ISA dataset [27]. For the ISA dataset, the frequencies of the clones were calculated using the number of the integration sites detected for each clone divided by the total number of integration sites detected in the samples analyzed (Table 2). Pearson’s correlation analysis shows a strong, positive relationship (*r* = 0.838; *p* = 0.037) between the two methods (Figure 3).

The clone frequencies calculated by IS-qPCR are generally higher than by deep ISA (Table 2). The reasons for this difference are not clear, but the two assays have very different methodologic approaches. The study by Halvas et al. used prior methods of identifying integration sites without specificity for individual proviruses, through population-based ISA [27,28], to estimate clone frequencies. IS-qPCR used the characteristics of the proviruses in specific clones to design each assay, including primers specific for both the adjacent host sequence and the proviral LTR and a probe specific for the host–virus junction. IS-qPCR also used a within-run external standard curve based on each host–virus junction to quantify the number of copies of a provirus detected as a fraction of total proviruses detected, also extrapolated from a within-run standard curve. The ISA assay has similar quantitative standards. Another possible explanation for the observed differences is that in estimating the total number of proviruses for the IS-qPCR calculations in Table 2, we presumed two LTR copies in each provirus. This assumption does not account for defective proviruses with deletions in one LTR, or more commonly solo LTRs, which could under-estimate the total number of proviruses in a sample and raise the calculated frequency of a specific clone. There may be other potential reasons to explain the discrepancy, such as proviral amplification efficiencies of both methods. We will continue to explore the differences in observed frequency through additional studies.

To investigate the potential off-target amplification, no template controls were routinely included in each assay run and no false positives were detected. In addition, each IS-qPCR primer and probe set was tested against genomic DNA extracted from two or three other HIV-1-positive donors. The results were the same as no template control samples, indicating no off-target amplification. Two proviruses (R-09 RAD50 and F-07 USP48) contain large internal deletions in *pol* through *env*, which truncate the proviral genomes to 3.2 kb. We considered the possibility that the PCR primers binding in the 3′ LTR could amplify through the shortened provirus, leading to an overestimate of the copy number. In the case of the R-09 RAD50 provirus, the binding site for the PCR primer is missing in the 3′ LTR, so priming from the 3′ LTR is not possible. For F-07 USP48, the binding site for the PCR primer in the 3′ LTR is approximately 2700 bp downstream from the 5′ LTR host–virus junction. We ran the product from several IS-qPCR wells on a gel and did not observe any dominant PCR product >700 bp (Supplementary Figure S1), indicating that PCR product from the 3′ LTR was unlikely to contribute to the calculation of the F-07 USP48 proviral copy number. In addition, sequence analysis of the IS-qPCR products matched that of the expected amplicon from the flanking host sequences to the 5′ LTR.

**Figure 4:**
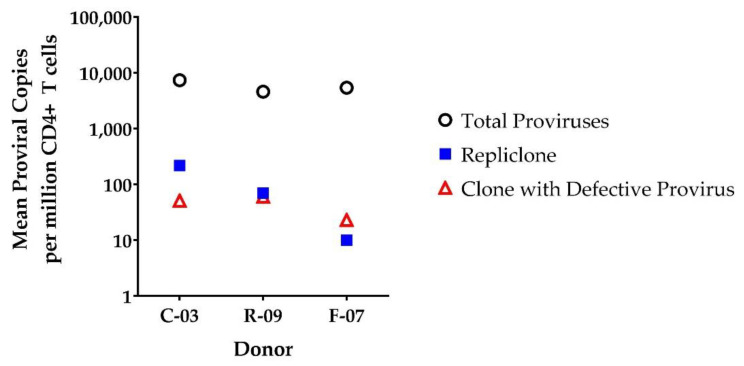
Numbers of proviruses in specific clones, calculated from IS-qPCR, relative to total provirus in all the infected cells. Values are reported as mean copies adjusted per million total CD4+ T cells.

To compare the relative sizes of the different clones determined by IS-qPCR, we plotted the estimated frequencies of specific clones and the estimated number of total infected cells in the samples from each donor (Figure 4). Using CCR5 measurements to estimate cell equivalents and the frequency of total CD4+ T cells previously reported for each donor [11], we determined the number of proviruses in each clone per 1 million CD4+ T cells. The sizes of the clones in Figure 4 were calculated using the average number of integration sites detected by IS-qPCR for the provirus of interest and the total number of proviruses detected by LTR-specific qPCR (correcting for two LTRs per provirus), both adjusted per 1 million CD4+ T cells. The number of proviruses per million total CD4+ T cells in each of the clones were estimated as follows: C-03 ZNF268, 218; C-03 BRCA1, 51; R-09 ABCA11P, 70; R-09 RAD50, 60; F-07 ZNF721, 10; F-07 USP48, 23. The average number of total proviruses detected per 1 million total CD4+ T cells ranged between 5404–7378 across the donors. The frequencies of the repliclones and the clones that carry defective proviruses analyzed were not markedly different (Figure 4).

## 4. Discussion

We describe here a novel and sensitive method to measure the frequencies of individual clones in genomic DNA from cells (PBMC or total CD4+ T cells) obtained from HIV-1-positive persons on ART. We first showed that the IS-qPCR assay is an accurate and sensitive method by measuring numbers of the major provirus in ACH-2 cells, which is integrated in the NT5C3A gene. The number of copies of the provirus integrated in the NT5C3 gene, as determined by IS-qPCR, was quite similar to the input cell equivalents (Table 1). Although off-target effects are possible with IS-qPCR, we did not detect off-target amplifications when the primers and probe designed for the NT5C3A provirus were used with genomic DNA from an HIV-1-positive donor or when used with no template controls. The results of IS-qPCR using DNA from a control donor were not different from no template control samples, indicating that no off-target amplifications were detected.

We also used IS-qPCR on clinical samples from HIV-1-positive donors in which the proviruses of interest are found among a background of uninfected host DNA and other proviruses. Previously published repliclone frequency data [11], which were based on a deep integration site sampling, were compared to clone frequencies measured by IS-qPCR (Table 2). To ensure a valid comparison of the methods, we analyzed the samples from the same time points using both methods (Supplementary Table S3). Overall, the clone frequencies obtained with the two methods were strongly correlated (*r* = 0.838, Pearson). The correlation is improved when the frequencies of the ABCA11P repliclone from donor R-09, which showed the greatest difference in frequencies (1.53% estimated by IS-qPCR and 0.03% estimated by ISA) are excluded (*r* = 0.989) (Figure 3). The agreement of the two assays is best highlighted by comparing the relative frequencies of the repliclones from C-03 and F-07, which show that the method can be used with either large (>1% of infected cells) or much smaller (<0.1% of infected cells) clones (Table 2). 

Despite high-level sampling by both methods, we observed as noted above, a discrepancy in the estimated frequency of the R-09 ABAC11P repliclone between the two methods (Table 2). There are several possible reasons for this discrepancy. The overall methodologic approaches differ: integration site-specific amplification and probe detection of the specific amplicon (IS-qPCR) vs. non-specific, host–virus amplification and sequencing to detect integration sites (ISA). In addition, different PCR primers and amplification conditions were used in the two assays. We considered the possibility that the primer and probe set used to detect the provirus integrated in ABCA11P falsely detected an off-target sequence. We tested the ABCA11P-specific IS-qPCR assay with genomic DNA from other HIV-1-positive donors; however, there was no detectable off-target amplification. We also sequenced amplicon products from different IS-qPCR reactions, which confirmed that PCR product was specific for the host–virus junction. It is possible that primers used for IS-qPCR that amplified the ABCA11P host–virus sequences were a better match for the specific proviruses than the ISA primers; however, more work and additional comparisons are needed to assess the difference in these results.

The estimated sizes of the repliclones from the three different donors range from 10 to 218 per million CD4+ T cells. The estimated sizes of the repliclones from C-03 and R-09 (218 and 70 per million CD4+ T cells, respectively) appear to be large in comparison to the absolute frequencies of intact proviruses recently reported using the IPDA, which measures total intact proviruses [25]. Specifically, Simonetti et al. reported a median of 54 intact proviruses per million CD4+ T cells among individuals on ART (*n* = 400), but with a 3-log range across individuals [25]. The largest of the three repliclones studied here (found in donor C-03) exceeds the reported median of 54 intact proviruses per million CD4+ T cells by 4-fold and comprises 3% of all infected cells. These data suggest this repliclone constituted a substantial fraction of the HIV-1 reservoir, but additional data for specific repliclones and total intact proviruses from these and other donors are required to draw conclusions about the contribution of individual repliclones to the HIV-1 reservoir.

The IS-qPCR workflow presented in this study has considerable promise as a tool that can be used to measure individual clones that comprise small, specific subsets of the HIV-1 reservoir. Two limitations of IS-qPCR are that it can only analyze a few proviruses at a time, and the integration site in the clone must be known. If the goal is to distinguish clones that carry defective and intact infectious proviruses, the sequence of the provirus is also needed. Characterizing the proviruses in clones, and their integration sites is a rate-limiting step. Until individual integrated proviruses can be characterized more rapidly, and on a larger scale, IS-qPCR will be limited to the proviruses in a small subset of clones. The current study is also limited by the small number of patient-derived proviruses that were used to validate the assay. More work is required to determine whether there will be additional discrepancies between the two assays.

There are several potential advantages of the IS-qPCR approach. When tracking a clone of interest, we have shown that, even for rare clones (<0.1% of infected cells, i.e., F-07 ZNF721), IS-qPCR requires only a few million cells. While low cell numbers represent a challenge in recoverable DNA extracted (e.g., cerebral spinal fluid and other tissue biopsies), the amount of sampling required will vary depending on the fraction of total cells in the sample that make up the clone. Deep integration site sequencing is a valuable tool that can be used to explore the overall proviral landscape and the fraction of the infected cells with a particular integration site [2,16,40,42,43]. In our experience, however, the recovery of integration sites is higher from purified CD4+ T cells than from PBMC, whereas IS-qPCR works equally well with PBMC and CD4+ T cells. The process of isolating total CD4+ T cells typically results in 5–20% recovery of the CD4+ T cells, which requires about five times the number of starting PBMC. IS-qPCR may be particularly advantageous when monitoring infrequent clones and cell numbers are limited. 

In conclusion, IS-qPCR has the potential to be used in several areas of HIV-1 research. IS-qPCR may serve to complement deep integration site sequencing or IPDA in characterizing the HIV-1 reservoir by measuring small populations of clonally-expanded cells. IS-qPCR could be applied longitudinally to measure frequencies of clones that carry intact and defective proviruses in T cell subsets in blood and in different tissue samples, depending on cell availability and clone frequency. In addition, IS-qPCR could be used, in place of QVOA or single-genome sequencing, to measure the response of specific repliclones and clones that carry defective proviruses to therapeutic interventions. The persistence of a stable HIV-1 reservoir represents a major challenge for curing HIV-1 infection. IS-qPCR has the potential to provide a deeper understanding of the dynamics of specific clones of infected cells on ART and to determine how individual HIV-1-infected clones respond to curative strategies.

## Figures and Tables

**Table 1:** IS-qPCR validation on ACH-2 genomic DNA.

ACH-2 Cell Eq.	Cell Eq. Detected ^1^	NT5C3A Provirus Copies Detected ^2^
100	87 ± 16	199 ± 14
30	31 ± 4	52 ± 13
10	10 ± 3	18 ± 7
3	3 ± 2	4 ± 1
1	0.7	2 ± 1
0.3	0.8	0.2 ± 0.2
^3^ HIV-1-positive PBMC	1,204,200	Not detected

^1^ Cell equivalents were determined by CCR5 quantification using qPCR (*n* = 3) ± intra-assay standard deviation. ^2^ NT5C3A provirus copies were determined using IS-qPCR (3 independent runs with 6 and 12 replicates) ± inter-assay standard deviation. ^3^ Genomic DNA extracted from an HIV-1-positive donor.

**Table 2:** Comparable clone frequencies in clinical samples determined by IS-qPCR and deep ISA.

Donor	Provirus	^1^ Total Cell Eq. Assayed by IS-qPCR	^2^ Clone Frequency (IS-qPCR)	^3^ Clone Frequency (Population IS Assay)	
C-03	ZNF268 (Repliclone)	5,604,300	2.98%	1.08%	44/4164
	BRCA1	4,736,700	0.68%	0.24%	10/4174
R-09	ABCA11P (Repliclone)	6,265,800	1.53%	0.03%	3/9939
	RAD50	3,861,000	1.32%	0.60%	60/9939
F-07	ZNF721 (Repliclone)	6,266,100	0.08%	0.05%	4/8407
	USP48	1,362,000	0.42%	0.10%	9/8315

^1^ Cell equivalents were determined by CCR5 quantification using qPCR [29]. ^2^ Clone frequencies were calculated using the fraction of the provirus of interest and the number of LTR copies (adjusted 2-fold to account for 2 LTR copies per provirus) measured by qPCR. ^3^ Clone frequencies calculated from the numbers of specific proviruses present in a clone or repliclone in a sample (numerator) and the total number of integration sites obtained from the same sample (denominator) using ISA [11].

## Data Availability

New proviral sequence data reported in this paper (i.e., C-03 BRCA1, R-09 RAD50, and F-07 USP48) have been deposited publicly in the GenBank database (accession numbers: MZ243211- MZ243213). Viral sequences and integration sites were also submitted to the Proviral Sequence Database (PSD; https://psd.cancer.gov/, accessed on 27 May 2021) [42] and the Retrovirus Integration Database (RID; https://rid.ncifcrf.gov/, accessed on 27 May 2021) [43] at the US Department of Health and Human Services, NIH, National Cancer Institute (Frederick, MD, USA).

## Supplementary Materials

The following are available online at https://www.mdpi.com/article/10.3390/v13071235/s1, Table S1: IS-qPCR Host-HIV-1 Primer Pair and Probe Sequences; Table S2 LTR Primer Pair and Probe Sequences used in qPCR; Table S3: Time Points of Donor Samples and Cell Types used for ISA and IS-qPCR; Table S4: Individual IS-qPCR Intra-Assay Results; Figure S1: Gel electrophoresis product from the F-07 USP48 host–virus junction; Table S5: Demographics and Clinical Histories of Donors Referred for Nonsuppressible Viremia; Table S6: Baseline Immunologic and Virologic Characteristics of Donors Referred for Nonsuppressible Viremia; Table S7: Reagents and Resources Suggested in IS-qPCR Workflow.
