# Supplementary material for: Tracking HIV-1-Infected Cell Clones Using Integration Site-Specific qPCR
Source: Viruses. 2021 Jun 25;13(7):1235. doi: 10.3390/v13071235 (PMC8310066; doi:10.3390/v13071235)
Supplement: Supplementary file 1 [file viruses-13-01235-s001.zip › viruses-1239128-supplementary.pdf]

Supplementary Table S1. IS-qPCR Host-HIV-1 Primer Pair and Probe Sequences.

Donor (Gene in which provirus is integrated)	Host-Specific (FWD) Primer Sequences (5′-3′) [Upstream Host-Virus Junction Position of Primer]	HIV-1 Specific (REV) Primer Sequences (5′-3′) [5′ Position of Primer]	IS-qPCR Probe Sequences (5′-3′)	Probe Strand Relative to Provirus
C-03 (ZNF268)	GAGTCTGGAATTGAATACATG [113]	ACAAATCAAGGATGTCTTGTCT [49]	CCAAACTAGCCCTTCCAAGTATACAGTGAT	Antisense
C-03 (BRCA1)	CAAATCCCAAGTCGTGTG [32]	CAGGGAAGTAACCTTGTGTG [78]	CTATATAACTGGAAGGGCTAGTTTGGTCCC	Sense
R-09 (ABCA11P)	TGCCTTTGTGCTTTAAGAAACTC [52]	GAAGTAGCCTTGTGTGTGGTAG [74]	AGATGGTATGTACCCTGGAAGGGCT	Sense
R-09 (RAD50)	ATCACATCTACCATTCTGGAC [39]	CGTTCTCTTCTTCTCCATCT [186]	ATCTCCTGGAAGGGCTAATTTACTCCC	Sense
F-07 (ZNF721)	GAATGCTTTACCACACAGTAC [26]	GTGTAGTTCTGCCAATCAG [94]	AGCTGGAAGGGCTAGTTTACTCCCAG	Sense
F-07 (USP48)	ATAAGGCAGGTATGTATTTGG [82]	GCCAATCAGGGAAGTAACC [84]	TAACTGTGAGATTGGAAGGGCTAG	Sense
ACH-2 (NT5C3A)	TGAGGAACAGATTTTCTCACATG [40]	AGATCAAGGATATCTTGTCTTC [46]	GAATGCTTTGTAATTGGAAGGGCTAATTC	Sense

Probes are labeled with a 5′ Fluorescein (6-FAM) fluorophore and two quenchers (3′ Dark Quencher and an internal ZEN quencher) (IDT) to reduce background fluorescence so the target region can be detected at low levels (see Methods).

Supplementary Table S2. LTR Primer Pair and Probe Sequences used in qPCR.

LTR (FWD) Primer Sequence (5′-3′)	LTR (REV) Primer Sequence (5′-3′)	LTR Probe Sequence (5′-3′)
GKGAACCCACTGCTTAAG	GTCTGAGGGATCTCTAGPTAC	TCAATAAAGCTTGCCTTGAGTG

Primer sequences were synthesized with internal modified bases, K and P, as published [1]. Probes are labeled with a 5′ Fluorescein (6-FAM) fluorophore and a 3′ Dark Quencher (NFQ) with a minor groove binder (MGB), which helps to form stable duplexes with the DNA target and increases binding specificity.

Supplementary Table S3. Time Points of Donor Samples and Cell Types used for ISA and IS-qPCR.

Donor	Date of Blood Draw	Cell Type (ISA)		Cell Type (IS-qPCR)	
		PBMC	Total CD4+ T Cells	PBMC	Total CD4+ T Cells
C-03	9/20/2014	X		X	
	11/30/2015	X		X	
	10/5/2017		X		X
R-09	8/6/2015	X	X	X	
	9/14/2016	X		X	
F-07	6/3/2015	X		X	
	4/6/2017		X		X

The clone frequencies of the repliclones presented in Halvas *et al.* 2020 [2] were generated from a compilation of several integration site sequencing assays (ISA) from multiple time points derived from a combination of PBMCs and total CD4+ T cells, denoted by X. To ensure the most accurate comparison of IS-qPCR, we sampled parallel cell types for each donor at each time point, when available (X).

Supplementary Table S4. Individual IS-qPCR Intra-Assay Results.

Donor	Provirus-Specific IS-qPCR Assay	Date of Blood Draw	Number of Replicates Positive for Provirus by IS-qPCR Assay [A]	Average Proviral Copies per Assay [B]	Standard Deviation of Assays [C]
C-03	ZNF268	10/5/2017	6/6	59.1	7.3
	BRCA1		6/6	3.8	2.4
R-09	ABCA11P	8/6/2015	4/4	6.8	1.0
	RAD50		2/2	4.7	2.9
F-07	ZNF721	4/6/2017	14/16	1.1	0.7
	USP48		4/4	2.6	1.7

[A] The number of PCR replicates, each replicate consisting of triplicate PCR reactions, in which the provirus of interest was detected (numerator) of the total replicates assayed (denominator).

[B] Average proviral copies across IS-qPCR replicate assays ([A]) in which each replicate consisted of the averages of triplicate PCR reactions.

[C] Standard deviation of IS-qPCR replicate assays, each consisting of triplicate PCR reactions.

Supplementary Figure S1. Gel electrophoresis of IS-qPCR product from the F-07 USP48 host-virus junction. The product size is as expected (166 bp) and amplification of the correct target sequence was determined by dideoxy chain termination sequencing (Sanger).

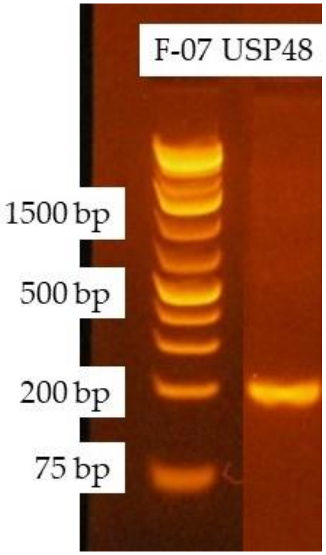

Supplementary Table S5. Demographics and Clinical Histories of Donors Referred for Nonsuppressible Viremia

Donor ID	Age	Sex	Race	Date of HIV-1 Diagnosis	Years On ART [A]	Year of Detectable Viremia [B]	Current ART Regimen [C]
**C-03**	43	Male	Caucasian	06/1994	9	4.5	DRV/r + ETV + DTG
**R-09**	73	Male	Caucasian	01/2005	10	5.2	TDF/FTC/EFV
**F-07**	59	Male	African American	06/1996	19	2.1	ABC/3TC + EFV

[A] Years on ART at the time of initial evaluation.

[B] Above the limit of detection of FDA-cleared plasma HIV-1 RNA assays.

[C] ABC, abacavir; DRV/r, darunavir/ritonavir; DTG, dolutegravir; EFV, efavirenz; ETV, etravirine; FTC, emtricitabine; TDF, tenofovir disoproxil fumarate; 3TC, lamivudine.

Tabled modified from [2].

Supplementary Table S6. Baseline Immunologic and Virologic Characteristics of Donors Referred for Nonsuppressible Viremia

Donor ID	Pre-ART Plasma HIV-1 RNA (cps/mL)	Nadir CD4+ T Cells (Cells/ mm^3)	Current CD4+ T Cells (cells/ mm^3)	Plasma HIV RNA at Referral (cps/mL) [A]	HIV-1 DNA (cps/ 10^6 PBMC) [B]	Cell-Associated HIV-1 RNA (cps/ 10^6 PBMC) [B]	IUPM [C]
C-03	16,700,000	10	416	62	2,505	1,162	1.4
R-09	97,000	105	380	197	1,533	139	18.1
F-07	117,068	314	1,023	52	1,603	1,112	3.8

[A] Plasma HIV-1 RNA copies/mL at time of referral determined by FDA-approved Roche CAP/CTM v2.0 or Abbott M2000 platforms.

[B] HIV-1 DNA and cell-associated RNA copies/million peripheral blood mononuclear cells (PMBC) measured by quantitative polymerase chain reaction [3].

[C] Infectious units per million (IUPM) total CD4+ T cells by quantitative viral outgrowth assay (QVOA) [4,5].

Table modified from [2].

Supplementary Table S7. Reagents and Resources Suggested in IS-qPCR Workflow.

	**Reagent**	**Resource**
**Multiple Displacement Amplification**	Phi29 DNA Polymerase and Reaction Mix	New England BioLabs
	Trehalose	Sigma-Aldrich
	$MgCl_2$	Fisher Scientific
	$(NH_4)_2SO_4$	New England BioLabs
	BSA	New England BioLabs
	Random 6-10-mer	IDT synthesis: phosphorothioate-terminated
	dNTP	New England BioLabs
	KOH	G-Biosciences
	EDTA	Fisher Scientific
	Trizma-HCl	Sigma-Aldrich
	Optimization considerations	[6,7]
**DNA Purification**	KAPA Pure Beads	Kapa Biosystems, Inc.
	GeneJET Gel Extraction Kit	Thermo Fisher Scientific
**Integrated Proviral Sequencing Assay ***	RANGER DNA Polymerase Mix	Bioline
	HIV primers FWD and REV	[2]
	Nullomer Adapter	[8]
	Optimization considerations	[9,10]
	SuperFi DNA Polymerase	Fisher Scientific
**Quantification of DNA**	1x TE buffer (5-10 mM Tris-HCl, pH 8.0, 1 mM EDTA)	Fisher Scientific
	Quanti-iT™ PicoGreen® dsDNA	Thermo Fisher Scientific
**Host-Full-Length_Host (HFH) Amplification**	RANGER DNA Polymerase Mix	Bioline
	HIV primers FWD and REV	Genewiz or IDT
	Complement Host Primers	Genewiz or IDT
**Amplification of qPCR Standard Template**	RANGER DNA Polymerase Mix	Bioline
	HIV REV Primer	[11]
	Host FWD Primer	HFH Nested Primer
**qPCR**	LightCycler® 480 Probes Master Mix	Roche
	IS-qPCR Host and HIV Primers	Genewiz or IDT

	IS-qPCR Probe	IDT
	CCR5 TaqMan Control Genomic DNA	Invitrogen
	CCR5 qPCR Primers and Probe	[3,12]

* Integrated Proviral Sequencing Assay detailed methodology in manuscript under review

1. Kong Lin Thoo, P.; Brown, D.M. Synthesis of oligodeoxyribonucleotides containing degenerate bases and their use as primers in the polymerase chain reaction. *Nucleic Acids Res.* **1992**, *20*, 5149–5152.
2. Halvas, E.K.; Joseph, K.W.; Brandt, L.D.; Guo, S.; Sobolewski, M.D.; Jacobs, J.L.; Tumiotto, C.; Bui, J.K.; Cyktor, J.C.; Keele, B.F.; et al. HIV-1 viremia not suppressible by antiretroviral therapy can originate from large T cell clones producing infectious virus. *J. Clin. Invest.* **2020**, doi:10.1172/jci138099.
3. Hong, F.; Jacobs, J.L.; Aga, E.; Cillo, A.R.; Fyne, E.; Koontz, D.L.; Zheng, L.; Mellors, J.W. Associations between HIV-1 DNA copy number, proviral transcriptional activity, and plasma viremia in individuals off or on suppressive antiretroviral therapy. *Virology* **2018**, *521*, 51–57, doi:10.1016/j.virol.2018.05.018.
4. Siliciano, J.D.; Siliciano, R.F. Enhanced Culture Assay for Detection and Quantitation of Latently Infected, Resting CD4+ T-Cells Carrying Replication-Competent Virus in HIV-1-Infected Individuals. *Methods Mol. Biol.* **2005**, *304*, 3–15, doi:10.1385/1-59259-907-9:003.
5. Rosenbloom, D.I.; Elliott, O.; Hill, A.L.; Henrich, T.J.; Siliciano, J.M.; Siliciano, R.F. Designing and Interpreting Limiting Dilution Assays: General Principles and Applications to the Latent Reservoir for Human Immunodeficiency Virus-1. *Open Forum Infect. Dis.* **2015**, *2*, ofv123, doi:10.1093/ofid/ofv12.
6. Pan, X.; Urban, A.E.; Palejev, D.; Schulz, V.; Grubert, F.; Hu, Y.; Snyder, M.; Weissman, S.M. A procedure for highly specific, sensitive, and unbiased whole-genome amplification. *Proc. Natl. Acad. Sci. U. S. A.* **2008**, *105*, 15499–15504, doi:10.1073/pnas.0808028105.
7. Leichty, A.R.; Brisson, D. Selective whole genome amplification for resequencing target microbial species from complex natural samples. *Genetics* **2014**, *198*, 473–481, doi:10.1534/genetics.114.165498.
8. Hampikian, G.; Andersen, T. Absent sequences: Nullomers and primes. *Pacific Symp. Biocomput. 2007, PSB 2007* **2007**, *366*, 355–366, doi:10.1142/9789812772435_0034.
9. Joseph, K.W.; Halvas, E.K.; Brandt, L.D.; Patro, S.C.; Rausch, J.W.; Chopra, A.; Mallal, S.; Kearney, M.F.; Coffin, J.M.; Mellors, J.W. Deep Sequencing Analysis of HIV-1 Proviruses Reveals Frequent Asymmetric Long-Terminal Repeats And Other Novel Proviral Structures. *Manuscr. Submitt.* **2021**.
10. Joseph, K.W.; Halvas, E.K.; Brandt, L.; Patro, S.; Rausch, J.; Chopra, A.; Kearney, M.; Coffin, J.; Mellors, J.W. Efficient High-Throughput Sequencing of Variable and Near Full Length Integrated HIV-1 Proviruses. In Proceedings of the Conference on Retroviruses and Opportunistic Infections (CROI), Boston, MA; Boston, MA, 2020; p. 127.
11. Li, B.; Gladden, A.D.; Altfeld, M.; Kaldor, J.M.; Cooper, D.A.; Kelleher, A.D.; Allen, T.M. Rapid Reversion of Sequence Polymorphisms Dominates Early Human Immunodeficiency Virus Type 1 Evolution. *J. Virol.* **2007**, *81*, 193–201, doi:10.1128/jvi.01231-06.
12. Malnati, M.S.; Scarlatti, G.; Gatto, F.; Salvatori, F.; Cassina, G.; Rutigliano, T.; Volpi, R.; Lusso, P. A universal real-time PCR assay for the quantification of group-M HIV-1 proviral load. *Nat. Protoc.* **2008**, *3*, 1240–1248, doi:10.1038/nprot.2008.108.
